# Monthly Summary

**Published:** 1872-11

**Authors:** 


### MONTHLY SUMMARY.

*The Influence of Marriage upon Health.*—M. Bertillon lately having had to draw up a paper for the Academy of Medicine of Paris on the influence of marriage on mortality, consulted the registers of the only three countries in Europe which were carefully enough kept to give him a reply to his question, those of France, Belgium, and Holland, and shows that if the male sex be first considered, we find that from 25 to 30, 1,000 married men furnish 6 deaths; 1,000 unmarried 10 deaths; and 1,000 widowers, 22 deaths. From 30 to 35, of 1,000 married men, 7 die; of 1,000 unmarried men, 11½ die; and of 1,000 widowers, 19 die. From 35 to 40, of 1,000 married, 7½ die; of 1,000 bachelors, 13 die; and of 1,000 widowers 47½ die; and so on all the following ages, the married man continues to live with greater facility than the bachelor. It has been said that since the most fortunate of men can afford to marry, it is not astonishing that these persons should live longer. But this will not of course, account for the very great mortality of widowers at all ages, which, indeed, surpasses that even of bachelors.

However, it must be noticed that 8,000 young men marry in France yearly, under the age of 20. This is very fatal to such young men, for M. Bertillon finds that, whilst 1,000 young men from 15 to 20 furnish 7 deaths, when unmaried, no less than 50 deaths occur among 1,000 young married men under 20. Hence *premature* marriage is the surest way to old age.

Women seem to reap less advantage from marriage than men, and there is but little difference in the mortality of unmarried and married women before the age of 25. It is but little marked even between the age of 25 and 30. From 30 to 35, out of 1,000 spinsters, 11 die; and of 1,000 wives 9 die. This difference augments up till 55 years of age. Thus, from 50 to 55 years of age, 1,000 wives have from 15 to 16 deaths a year, whilst 1,000 spinsters or widows have 26 to 27 deaths. This advantage rests very marked beyond this age, although it diminishes a little; but before 25 years of age in France, and before 20 in Paris, marriage, far from being favorable to the life of women, is injurious to it. The mortality of maidens from 15 to 20 is 7.53 per 1,000; of wives of the same age, is 11.86 per 1,000; and the mortality of married women from 20 to 25 being 9.92 per 1,000, the mortality of maids of the same age is 8.32.

With respect to widowhood, from 25 to 30, widowhood is injurious to health; and whilst 1,000 wives and maids of this age furnish 9 deaths a year, 1,000 widows have 17 deaths.

But in France, and especially at Paris, this mortality quickly diminishes, and at 45, the death-rate of widows is lower than that of spinisters at the same age. And at this age women who have had children are least attacked by death. "Thus marriage in women retards old age, and soothes its sufferings."

The conclusions of these statistics is that marriage is an element of health much more powerful than has ever been supposed, that it exercises its salutary influences especially on the male in the age of vigor, and on the female in her declining years. A calculus of probabilities shows that the man who marries between 20 and 25 has 40 years to live in place of 35, and that the young woman who marries between 20 and 25 has 40 in place of 36. Criminality, suicide and insanity are far less common among the married than the single.—*Druggists' Circular.*

---

*A Pill for the Doctors.*—George Washington was killed by his physicians by blood-letting; Prince Albert, the husband of England's queen, was killed also by his physicians with brandy; and the Prince of Wales had recently a very narrow escape from being killed by the same remedy; fortunately, at the nick of time, the brandy was given up, and then he got better. Charles Weston, the former publisher of of this journal, was killed by his physician by means of the injudicious administration of sulphate of quinine, persisted in to the last, notwithstanding our earnest protest; and we could go on in making a long list of well-known public characters and of many of our personal acquaintances, who when threatened to die by a disease, called in a doctor, who by injudicious prescriptions made a sure thing of what before was doubtful. The great secret of successful medical practice is to prescribe in such a way as not to do the patient any harm; this is the honest opinion of the most eminent old experienced physicians, and it reminds us of an incident in our own life, the communication of which may be amusing and instructive to our readers.

When, some fifteen years ago, we were filling a professorship in the New York Medical College, and practicing medicine in the city of New York, we visited often old Dr. Valentine Mott, then an octogenarian, but now deceased since some years. It was a delight to converse with the old gentleman, and see his eagerness for information in the newest discoveries in the field of electricity, which we were happy enough to supply. At one of these occasions he saw our medical pocket-case with small bottles, which at that time we always carried in order to be ready for any medical emergencies in the night. He exclaimed—

"Oh doctor! I hope you have not turned homœopath?"

"Not at all, Dr. Mott; these are the regular, allopathic remedies,"

"That is right, it would grieve me to see you fall so low; but why these homœopathic bottles?"

"In order to have a druggist's shop in a small compass, and not to be obliged to send to a druggist when called out at night."

"Well, that is right; but let me see your selection."

Then a critical examination of every bottle took place, and the choice approved as very judicious, till the last bottle was reached without label, containing white pills, when Dr. Mott said—

"And what is there in this bottle?"

We hesitated in our answer, but at last told the truth, half ashamed—

"I have to confess to you, Dr. Mott, that these are plain sugar pills."

"Those are the best pills yon have in your whole box," was the answer of the old venerable apostle of Æsculapius.—*Manufacturer and Builder.*

---

*Sick Headache.*—The true cause of sick headache lies deep in the patient's idiosyncrasy, and is developed by a hundred different causes. The advice, then, to sufferers is to give as much tone as they can to their nerves by adopting all those methods which experience has shown to be good, and then avoid, as far as practicable, all those causes which are known to excite an attack. I need scarcely describe a sick headache—how one rises in the morning more dead than alive; perfectly unable to swallow the smallest particle of food, and often perhaps actually sick; how the head throbs, and the pain increased by the slightest movement; how speaking or doing is a burden beyond bearing; how one prays to be left alone in the utmost quiet, so that he may, if possible, sleep. To other persons the sufferer looks extremely ill, very pale, dark around the eyes, and with contracted pupil. To himself his head feels hot, and the application of cold is more refreshing. The clamminess in the mouth, the nausea and general gastic disturbances are secondary, and have no connection with any improper meal, and thus is in no way relieved by the too frequent and ignorantly administered purgative. This is not needed, and has no good result. The only remedies which are of any avail are those which act on the nervous system, such as hot tea and coffee; or, after the stomach is quieter, and the more urgent symptoms have passed off, a little wine or ammonia. If the headache take more the form of hemicrania, then remedies are occasionally useful, as the local application of the bisulphide of carbon, or galvanism, and internally the bromide of potassium. This is the only drug which I have really seen to be serviceable. Whilst the nausea exists and the worst symptoms prevail, even this remedy is of no avail. —*British Medical Journal.*

*Subcutaneous Injection of Morphia and Chloroform.*—In *El Progreso Medico*, April 1st, we see it mentioned that Dr. Nussbaum has, from a desire to lessen the dangers of chloroform by diminishing the dose necessary to obtain anæsthesia, made use of subcutaneous injection of morphia simultaneously with chloroform inhalation. The combination of the two substances, it seems, has been studied by Claude Bernard with good results, and has been applied to surgery, and also in obstetrics. From experiments made by MM. Labbe and Goujon, of which an account was given to the Academy of Sciences (*Gas. Med. de Paris,*) it results that by making an injection of small doses of morphia before giving chloroform, we obtain a far longer sustained anæsthesia, with very little chloroform; and it is certain that the dangers of this medicament are in direct relation with the quantity breathed by a patient. This is a great step says the *Progreso Medico*, since great risk is spared to the patient, whilst at the same time the local and general sensibility of the patient is lessened. —*The Doctor.*

---

*Death from the Presence of a Piece of Needle in the Heart.*--The *Cincinnati Lancet and Observer* states that at the post mortem examination of Miss Hoag, Evanston, Ill., it was found that death was caused by the presence of a piece of needle in the heart. The needle was driven in by a sudden fall when she was about seven years old. The portion of needle extracted was about an inch and one-eighth long and of large size The editor remarks, "How life could have existed after it entered the heart, is a mystery even to medical men."

---

*Morphine as a Local Anœsthetic.*—Dr. Spessa states, in the *Bulletin des So. Med.* (Italy,) that he has succeeded in preventing pain, during the slitting of a fistulous tract, by injecting a solution of morphia into the tract before the use of the knife. The same author had occasion to touch the vulvar vegetations of a girl with butter of antimony ; the pain was very acute, but disappeared on the part being brushed over with a solution of morphia. A boy of fifteen, suffering from hip-joint disease, required an issue over and beyond the great trochanter. An injection of morphia was first made over the region, and Vienna paste applied, which latter remained about eight minutes. The paste did not give any pain. Dr. Spessa states that he would be glad to hear that a fair trial has been given to this mode of using morphia.—*London Lancet.*

---

*Deaths by Inhalation of Ether.*—To the facts already published, it is necessary to add three new cases. In one (Dr. Page) a young girl died during anæsthesia by ether, administered to arrest epileptic convulsions. In the second and third cases (Austin Martin,) it was for amputation of the leg. and a patient suffering from delirium tremens. Finally, a fourth case is reported by M. A. Martin, but the ether has acted in a very unusual manner; the surgeon, by an inexcusable folly, if not by ignorance, applied the actual cautery to the tongue of a patient under the influence of ether. The vapor of ether becoming inflamed caused a subacute bronchitis by burning. The patient died.—*Gazette Hebdone.*

---

*Hypertrophy of the Tongue.*—(G. Maas: *Arch. f. Klin. Chirurgie*, xiii., B. 3, H,)—In the surgical clinic in Breslau, during the past year, there were five cases of hypertrophy of the tongue reported. They were all congenital in their origin; the tongue was, in some instances, affected on one side only; in others, throughout its entire structure. In one case the hypertrophy of the left half of the tongue was accompanied by hyperplasia of the entire left half of the body. All the cases were treated by means of the galvano-caustic. The microscopical investigation in one case, two years old, revealed complete hyperplasia of all the tissues of the tongue, and, in three others, new growths of connective tissue and vessels, so that the tongue was completely metamorphosed into a spongy, cavernous mass.

The least amount of tissue-changes was in a patient three years old; a greater amount in one of twelve years, and the most prolific changes had occurred in a patient twenty-one years old.

Hence the conclusion that every case of macroglossa first consists of hyperplasia, to which is added increase in connective tissue and vessels, as a secondary result of continual irritation, caused by the enlarged and protruding tongue.—*Med. Times.*

---

*Anchylosis of the Inferior Maxilla Cured by the Formation of a False Joint on Each Side.*—(G. Maas: *Arch. f. Klin. Chirurgie*, xiii., B. 3, H.)—A man, 27 years old, had had in his seventh year an attack of scarlatina, which left him with an inflammation of the maxillary articulation on both sides, which was followed by complete anchylosis. Both the inferior and superior maxillary bones had suffered in consequence an arrest of development. Middeldorpf excised on one side, and subsequently Fischer on the other, a wedge-shaped piece from the inferior maxilla, the base of the wedge being downwards, according to Esmarch. The mobility had remained perfect at the time of the report, four months after the operation.—*Med. Times.*

